# DBHR: a collection of databases relevant to human research

**DOI:** 10.2144/fsoa-2021-0101

**Published:** 2021-01-20

**Authors:** Shahid Ullah, Wajeeha Rahman, Farhan Ullah, Gulzar Ahmad, Muhmmad Ijaz, Tianshun Gao

**Affiliations:** 1 Research Center, The Seventh Affiliated Hospital of Sun Yat-sen University, Shenzhen, Guangzhou, China; 2 S Khan Lab Mardan, Khyber Pakhtunkhwa, Pakistan

**Keywords:** DBHR, DNA database, human database, human genome, protein database

## Abstract

**Background:**

The achievement of the human genome project provides a basis for the systematic study of the human genome from evolutionary history to disease-specific medicine. With the explosive growth of biological data, a growing number of biological databases are being established to support human-related research.

**Objective:**

The main objective of our study is to store, organize and share data in a structured and searchable manner. In short, we have planned the future development of new features in the database research area.

**Materials & methods:**

In total, we collected and integrated 680 human databases from scientific published work. Multiple options are presented for accessing the data, while original links and short descriptions are also presented for each database.

**Results & discussion:**

We have provided the latest collection of human research databases on a single platform with six categories: DNA database, RNA database, protein database, expression database, pathway database and disease database.

**Conclusion:**

Taken together, our database will be useful for further human research study and will be modified over time. The database has been implemented in PHP, HTML, CSS and MySQL and is available freely at https://habdsk.org/database.php.

## Lay abstract

We have compiled the most recent collection of human research datasets into six categories – DNA database, RNA database, protein database, expression database, pathway database and disease database – on a single platform. In all, 680 human datasets were acquired and incorporated from scientifically published studies. There are several ways to retrieve the data, as well as original links and short descriptions for each database. The primary goal of our research is to store, organize and exchange data in an organized and searchable format. In brief, we have planned the future development of additional features in the database. Our database will be beneficial for future human research studies and will be updated throughout time. We firmly believe that every researcher should have access to essential biological databases, so we have gathered a collection of human-related databases that are regularly used and currently available but have not previously been presented in such a simple and welcoming manner.

## Introduction

Biological databases are libraries of life sciences information that provide access to genomic data [1–3] and analysis of genetic diseases, genetic genealogy or genetic fingerprinting for criminology [4], physical, chemical and biological information on sequence, domain structure, function, three-dimensional structure and protein–protein interactions [5,6], relationships between medical conditions, symptoms and medications [7], and information on cell signaling pathways [8], representing a great contribution by the scientific community. Many databases have been published in this research area, including the Kyoto Encyclopedia of Genes and Genomes pathway [9] BiGG Models [10], Database Commons, MiST 3.0 [11] and Pathway Commons databases [12], which are databases that contain biological pathways for metabolic, signaling and regulatory pathway analysis. The DNA Data Bank of Japan [13], GenBank [14], the European Nucleotide Archive [15] and Circadian Gene DataBase (CGDB) [16] are DNA databases that can be used for the analysis of genomic information [17], such as genetic diseases, genetic genealogy or genetic fingerprinting for criminology. The Eukaryotic Linear Motif database [18], Protein Data Bank in Europe [19], Database of Phospho-sites in Animals and Fungi [20] and the Conserved Domains Database [21] are protein databases that have been constructed from physical, chemical and biological information on proteins’ sequence, domain structure, function, three-dimensional structure and protein–protein interactions. miRTarBase [22], RNA Central [23] and NONCODE [24] include a huge group of eukaryotic RNAs involved in the regulation of gene expression. CancerGeneNet [25], Online Mendelian Inheritance in Man [26] and The Cancer Genome Atlas [27] provide information about the relationships between medical conditions, symptoms and medications. Expression Atlas [28], ArrayExpress [29] and BioExpress [30] are expression databases constituting an international public repository that archives and freely distributes high-throughput gene expression and other functional genomics datasets. Biological databases contain large quantities of omics data; according to the 2020 Molecular Biology Database Collection study in the journal *Nucleic Acids Research*, a total of 1637 databases are publicly accessible online [31–33], with a broad classification range. Several articles have been published in well-known journals relating to different organisms and components, such as the collection of 74 databases listed by Zou *et al*. [34], Previously, we gathered and published 59 COVID-19-related databases [35]. A comprehensive collection of the human databases is needed for the research community. Therefore, for more general-purpose and easy access, we have collected all the commonly used and currently available human databases to one platform, DataBases relevant to Human Research (DBHR) in which users can get the required category via a single click; for example, if a user needs a DNA database, they can directly get all 126 DNA databases on a single click and can choose the needed database. This is easier than searching each one via Google, and only updated database links have been provided (in the form of a table) [34,36–39]. As database classification based on data type is insightful, we allocate one major category to each database, although a single category can lead to multiple databases. The emphasis is on databases classified as DNA database, RNA database, protein database, expression database, pathway database and disease database. A comparison table of our work with previously published literature is shown in Table 1, which includes the category of the published work number of databases, the form of the data, PubMed reference number, year of publication and journal name. Furthermore, the DBHR can be explored in three ways: it can be searched either by clicking on the name or on the picture or by entering the name of the database in the search bar.

**Table 1.** Comparison of DBHR with other published work.

PMID	Year	Category	Type of database	No. of databases	Journal name	Ref.
**DBHR**	**2022**	**Human**	**DB + table**	**680**	–	–
34604832	2021	COVID-19	DB + table	59	*Computer Methods and Programs in Biomedicine Update*	[35]
25712261	2015	Human	Table	74	*Genomics, Proteomics and Bioinformatics*	[34]
18265344	2004	Protein	Table	121	*Current Protocols in Molecular Biology*	[40]
16381921	2006	Pathway	Database	190	*Nucleic Acids Research*	[41]
7764641	1994	DNA + Protein	Table	50	*Current Opinion in Biotechnology*	[42]
31906604	2020	Nucleic acid	Table	70	*Nucleic Acids Research*	[32]

## Materials & methods

### Construction of DBHR

**Figure 1.**
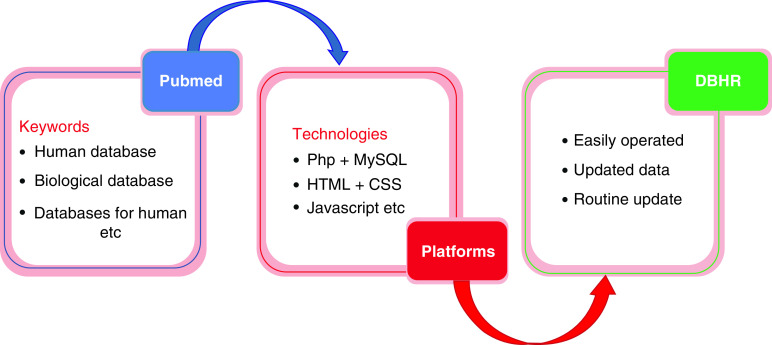
Procedure for the collection of the human databases data and construction of DataBases relevant to Human Research. DBHR: DataBases relevant to Human Research.

In this study we mainly focused on the collection of human databases. To avoid missing data, we used several keywords in PubMed [43] for example, ‘human database’, ‘biological databases’, ‘database for human’ and have combined each and every category with major keywords such as ‘human protein databases’, ‘human DNA databases’ and so on (Figure 1). We also manually collected the latest human databases from the journal Nucleic Acids Research [44], which is the cutting-edge research journal on databases. After removing broken links, programming platforms including PhP, MySQL, HTML, CSS and JavaScript were used to construct DBHR (Figure 1). By this method, we have provided a comparable human research database to the scientific community that is easy to operate and will be updated over time.

### Database classification

Many articles have been published in well-known journals (Table 1) [32,40–42,45–47], which have collected databases relating to different organisms and components. For example, Zou *et al*. [34] collected and published a list of 74 human databases in the journal Genomics, Proteomics and Bioinformatics, and Prakash *et al*. [48] collected a list of 24 fungi databases which was published in the Journal of Clinical Microbiology. However, a comprehensive human database is also needed for the research community to sort and save all the human data for future researchers. Further, published work has collected the databases and has presented them in the form of a table, while in our work we have provided the table as well as the database of the databases, and have the comparison table (Table 1) which shows a clear improvement. According to the diverse purposes of biological databases and published literature [34,49–51], we have classified the human-related biological databases into the following six categories.

#### DNA databases

The DNA databases provide access to genomic data contributed by the scientific community from more than 900 species whose sequencing and mapping is either completed or ongoing. There are now more than 57 completed microbial genomes and 245 reference sequences for eukaryotic organelles available in different DNA databases [1]. DNA data can be used for the analysis of genetic diseases, genetic genealogy or genetic fingerprinting for criminology [4]. Some databases allow for the management of DNA data from specific species [34], such as DNA Data Bank of Japan [13], GenBank [14], the European Nucleotide Archive [15] and CGDB [16].

#### RNA databases

It is well known that only a limited amount of the human genome is transcribed into mRNAs, while the vast majority of the genome is transcribed into noncoding RNAs that do not code for proteins, these include microRNAs, nucleolar RNAs, piwiRNAs and long noncoding RNAs [34]. An example of an RNA database is the microRNA database miRBase, which was first released in 2002 and is currently the most complete resource for information on microRNAs, a diverse group of eukaryotic RNAs involved in the regulation of gene expression.

#### Protein databases

A protein database is a collection of data that has been constructed from physical, chemical and biological information on proteins’ sequence, domain structure, function, three-dimensional structure and protein–protein interactions [52]. The purpose of the protein databases is to arrange and annotate protein structures, providing the biological community with valuable access to experimental evidence, an example is the Protein Data Bank [53]. Published scientific databases such as Antibodies Chemically Defined [54], the Plant Protein Phosphorylation Database[55] and the Structural Classification of Proteins database [56] are well-known databases in the protein research area.

#### Disease databases

Disease databases provide information about the relationships among medical conditions, symptoms and medications [7]. Comprehensive disease classification, integration and annotation are crucial to biomedical discovery. There is a variety of well-known and referenced databases that include a set of human genes and genetic phenotypes [57], including The Cancer Genome Atlas [27] and the International Cancer Genome Consortium data portal [58].

#### Pathway databases

Pathway databases contain biological pathways for metabolic, signaling and regulatory pathway analysis. Several databases contain information on cell signaling that has been developed in accordance with data access and analysis methodologies [8] and have been published in this research area. Examples include the Kyoto Encyclopedia of Genes and Genomes pathway database [9], BiGG Models database [10], MiST 3.0 [11] and Pathway Commons [12].

#### Expression databases

The Gene Expression Omnibus database is an international public repository that archives and freely distributes high-throughput gene expression and other functional genomics data sets [59]. Translation makes it easier to understand biological processes under normal or disease-related conditions. Researchers trying to identify similarities and differences between organisms at the molecular level need resources to collect data on multi-organism tissue expression [60].

## Results & discussion

### Statistics of DBHR

**Figure 2.**
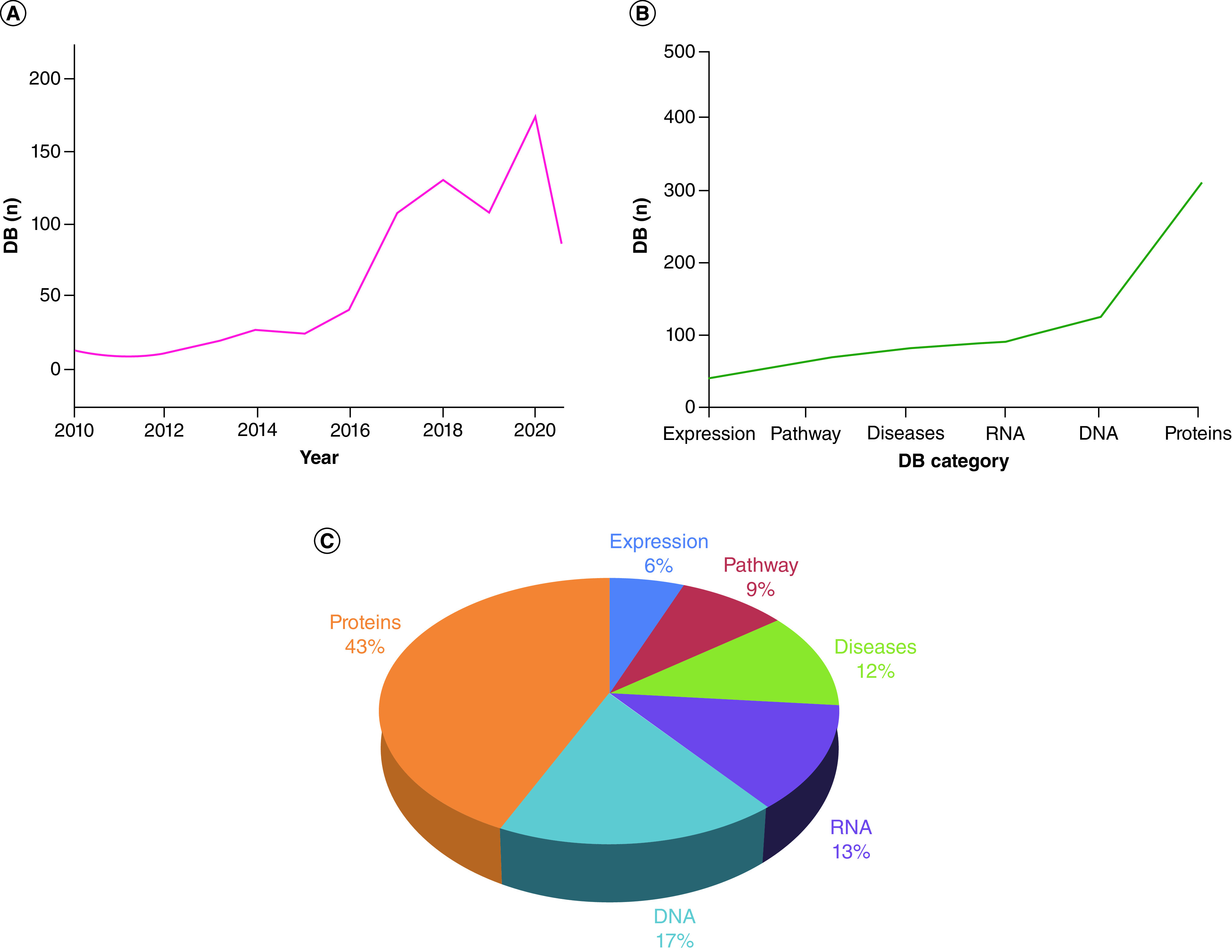
The statistics data of DataBases relevant to Human Research. **(A)** Chronological order of the DBHR. **(B)** Category-wise growth of the DBHR. **(C)** Distribution of the database categories. DB: Database; DBHR: DataBases relevant to Human Research.

In this study we have curated the year-wise and category-wise databases, have modified or deleted all the outdated, broken and non-assessable database links (Supplementary Table 1), and have provided new and updated human databases (Supplementary Table 2), thus demonstrating the rapid growth of biological databases (Figure 2A). In addition, the category-wise development of the DBHR is demonstrated by the different data categories (Figure 2B) which represent tremendous growth and achievement for the scientific community, due to the rapid growth of these results. Figure 2C shows the distribution of the categories as percentages.

### Usage of DBHR

**Figure 3.**
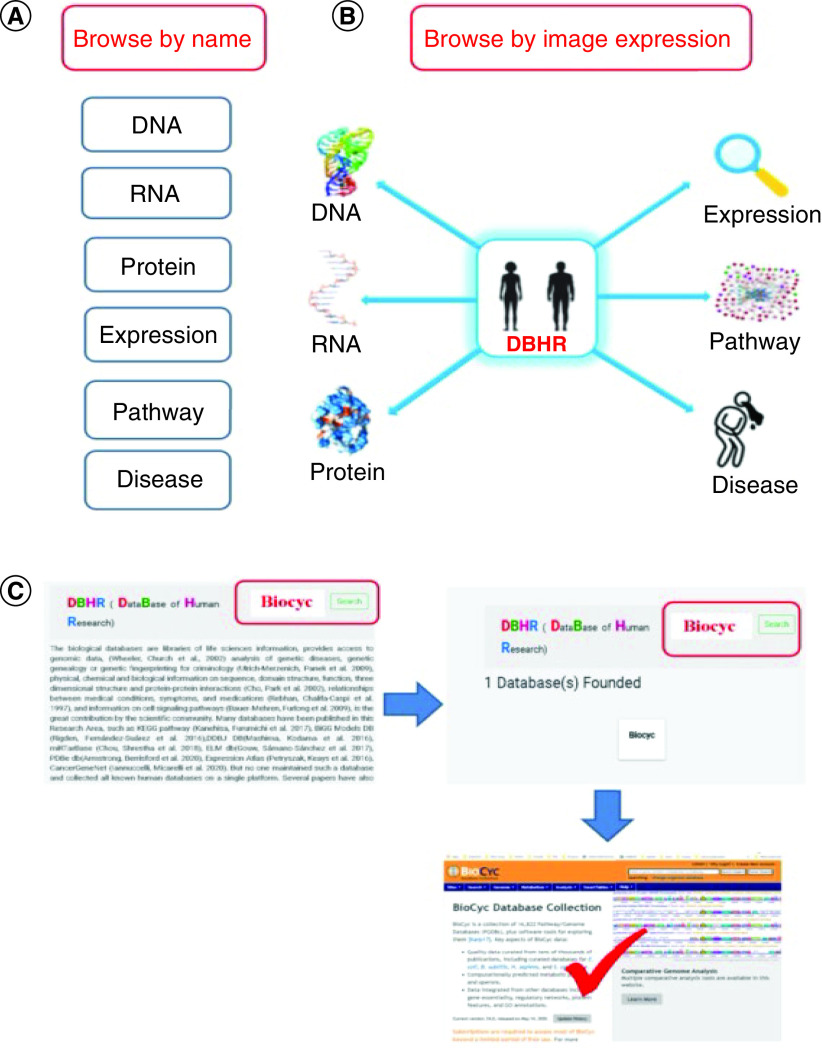
The browsing options of the DataBases relevant to Human Research. **(A)** Browsing by clicking the name. **(B)** Browsing by clicking the image. **(C)** Browsing using the search bar.

The DBHR has been developed to make searching easy and user-friendly. For easier and faster searching, three options are provided for finding a human database. First, DBHR can be browsed by the name of the category (Figure 3A) or related image (Figure 3B), a new feature of accessing the database that has not been provided before in such database fields. This search will lead to the category list page, and a brief overview with the original link of the required search will be accessed by clicking the needed database. Further, for database search, users can enter the name of the required database in the search bar (Figure 3C). In Figure 3C the BIOCYC database is used as an example from the disease databases to make it easier for users, some relevant work is shown in Table 2.

**Table 2.** Some relevant research work in the field of databases and resources.

Type of study	Study (year)	Summary	Ref.
Plant	Ullah *et al.* (2021)	Collected all the plant-related databases	[47]
COVID-19	Ullah *et al.* (2021)	Provide an innovative and user-friendly platform for COVID-19 research	[35]
Human	Zou *et al.* (2015)	Presented a collection of human-related biological databases and provided a mini-review	[34]
Protein	Xu (2012)	Protein databases on the internet	[40]
Pathway	Bader *et al.* (2006)	Pathguide: a pathway resource list (comprehensive study on pathway databases)	[41]
DNA + protein	Harper (1994)	DNA and protein databases and resources.	[42]
Nucleic acid	Rigden *et al.* (2016)	Database issue of *Nucleic Acids Research* and an updated molecular biology database collection	[6]

## Conclusion

The main objective of our study was to store, organize and share data in a structured and searchable manner, with the aim of facilitating the retrieval and visualization of data for humans. We strongly believe that every researcher should have access to important biological databases, we are therefore bringing together a set of human-related databases that are commonly used and currently available and have not previously been published in such an easy and user-friendly way. As database classification based on data type is insightful, we allocated one major category to each database, although a single category can lead to multiple databases. The emphasis is on databases classified as DNA database, RNA database, protein database, expression database, pathway database or disease database. We provided access to 680 up-to-date human databases in a fast, easy and user-friendly way, DBHR can be searched either by clicking on the name of the category or the category image, and also by entering the name of the database in the search bar. The facility will be upgraded with the passage of time.

## Future perspective

According to the huge and rapid increase of human-related research databases, which cannot be handled without computational databases, and is rapidly becoming a critical component of modern biology. In any case, database research is always the initial step in all biological study, nevertheless, the utilization of multiple databases also aids researchers in understanding the evolution, structure, and function of all proteins. However, for further research, a comprehensive and large-scale database is required. As a result, as time passes, we will strive to deliver the most up-to-date human research databases with more specific categorization to the scientific community. Furthermore, as science progresses, we will offer some advanced searching in the near future.

**Summary points**

- Our facility, DBHR (DataBases relevant to Human Research) aims to provide useful insights for researchers with the gathering of all relevant human data to one platform.
- DBHR provides access to data from sources that are difficult to locate.
- DBHR gives details that may not have been published before in such an easy and user-friendly way in the open literature.
- DBHR also monitors and updates dead and broken databases to ensure that only current information is presented.
